# Potential Antioxidant and Angiotensin I-converting Enzyme Inhibitory Activity in Crust of Dry-aged Beef

**DOI:** 10.1038/s41598-020-64861-0

**Published:** 2020-05-12

**Authors:** Juhui Choe, Bumjin Park, Hyun Jung Lee, Cheorun Jo

**Affiliations:** 1 Department of Agricultural Biotechnology, Center for Food and Bioconvergence, and Research Institute of Agriculture and Life Sciences, Seoul National University, Seoul, 08826 Republic of Korea; 2 Institute of Green Bio Science and Technology, Seoul National University, Pyeongchang, 25354 Republic of Korea

**Keywords:** Peptides, Metabolomics

## Abstract

Antioxidant activity, angiotensin I-converting enzyme (ACE) inhibitory activity, and protein profile of crust (the dried surface of dry-aged beef) were evaluated compared to unaged, wet-, and dry-aged beef. Antioxidant activity was determined using 2,2-diphenyl-1-picrylhydrazyl and 2,2′-azino-di-(3-ethylbenzthiazoline sulfonate) radical scavenging activities, ferric reducing antioxidant power, and ferrous ion chelating activity. The crust samples showed the greatest (*P* < 0.05) ACE inhibitory and antioxidant activity resulting from the three different mechanisms of action (radical scavenging, non-radical redox potential activity, and metal chelating) among the treatments. Small molecular weight protein bands and small peptides (<3 kDa) indicating potent bioactivity were evident in the myofibrillar protein profile of crust samples. The lowest (*P* < 0.05) ACE inhibitory activity was observed in unaged beef. The results indicate that crust could be utilized in various areas as a functional ingredient possessed antioxidant and ACE inhibitory activity instead of being discarded. In addition, dry aging can use for generation of functional ingredient from beef as the regime.

## Introduction

Postmortem aging enhances the tenderness and juiciness of meat through the activation of proteolysis by endogenous proteolytic enzymes^1^. The two methods of aging include wet aging (stored in vacuum packaging) and dry aging (stored under controlled temperature, relative humidity and airflow with exposure to air)^2^. During dry aging, air exposure in meat leads to considerable weight loss (20–45%) due to moisture evaporation and the generation of crust (trimming portion) on the meat surface that has intensively concentrated beefy flavor compared to wet-aged meat^3,4^. During dry aging, protein decomposition or concentration of free amino acids by microbial proteases or moisture evaporation respectively could bestow bioactivities, including antioxidant and angiotensin I–converting enzyme (ACE) inhibitory activities^5^. Recent studies reported the generation of several bioactive peptides with antioxidant and ACE inhibitory activities from meat and meat products during dry fermentation or aging with protease treatmen^6–8^. Muscle-based proteins contain unique amino acids with high bioavailability, such as methylhistidine and hydroxymethyl lysine^9^ and functional dipeptides including carnosine (β-Ala-His) and anserine [β-Als-His(3-Me)], which exhibit antioxidant activity as chelating metal ions or scavenging free radicals^10,11^. However, functional properties, including antioxidant and ACE inhibitory activity, of the crust produced by the dry aging process, are unknown and not studied. The crust accounts for approximately 35% of dry-aged meat, and is usually discarded.

We hypothesized that the crust which is inevitably produced from dry-aged meat possesses antioxidant and ACE inhibitory activities by the production of peptides during aging. This study determined the antioxidant and ACE inhibitory activities of the crust (trimmed portion of dry-aged beef) compared to unaged (fresh), wet-aged, and dry-aged beef samples. Furthermore, the difference in protein profiles was determined between unaged beef and crust samples and the identification of small peptides was conducted.

## Methods

### Meat sample preparation

At 24-h postmortem, loins (M. *longissimus*, n = 4) were obtained from 23-month-old castrated Holstein bulls (quality grade 3, n = 2) slaughtered locally. Each loin was cut into two sections and the six of eight sections randomly assigned to three treatments: (1) unaged (fresh), (2) wet-aged in vacuum packaging for 28 days at 4 °C, or (3) dry-aged for 28 days at 4 °C, RH of 75%, and airflow of 2.5 m/s. Crust was trimmed approximately 1 cm from the external surface of each dry-aged meat by a professional butcher. Pathogens were not detected in the crusts based on 16S rDNA sequencing (data not shown). All samples (crust, flesh of dry-aged, wet-aged, and fresh beef) were trimmed of visible fat and connective tissue, cut into cubes, and minced in a blender (HMF-985, Hanil, Incheon, Korea) for 30 s. The blended meat samples were immediately frozen in liquid nitrogen and lyophilized. The lyophilized samples were ground to a fine powder using a mortar and pestle, screened through a 10-mesh sieve, and kept at −70 °C until use.

### Extraction

The whole muscle protein was extracted from the beef samples with a minor modification and used for the further analysis of antioxidant and ACE inhibitory activities^12^. Briefly, 1 g of each beef sample was homogenized for 30 s in 10 mL of 0.02 M phosphate buffer (pH 7.4). The homogenate was centrifuged (Continent 512R, Hanil) at 1,500 × *g* for 15 min at 4 °C. The supernatant was collected and used for analysis of antioxidant and ACE inhibitory activities.

### Measurement of antioxidant activity

#### 2,2-Diphenyl-1-picrylhydrazyl (DPPH) assay

The meat extract was diluted 50 times with deionized distilled water (DDW), and 2 mL of the diluted samples were mixed with 2 ml of 0.2 mM DPPH in methanol^13^. After vigorous vortexing, the mixture was left in dark for 20 min at room temperature then centrifuged (Continent 512R, Hanil) at 2,268 × *g* for 10 min at 4 °C. The absorbance of the supernatant was measured at 517 nm using a spectrophotometer (X-ma 3100; Human Corp., Seoul, Korea). Trolox was used as the antioxidant standard. The absorbance was converted to the equivalent activity of Trolox per mL based on the standard curve and expressed as Trolox equivalent antioxidant capacity (TEAC, TEAC/ml extract). DPPH radical scavenging activity (%) was calculated as [1 − (Absorbance of sample at 517 nm/Absorbance of control at 517 nm)] × 100.

#### 2,2′-Azino-di-(3-ethylbenzthiazoline sulfonate) (ABTS) assay

ABTS capacity of each muscle extract was measured by the degree of suppression of ABTS radical cation (ABTS.+) produced by the reaction of ABTS with potassium sulfate^14^. To obtain the ABTS. + solution, a mixture of 7.0 mM ABTS and 2.45 mM potassium persulfate (final concentration) was prepared and stored in the dark for 16 h. The solution was diluted with ethanol to an absorbance of 0.70 ± 0.02 at 734 nm. Fresh ABTS.+ solution was prepared for each analysis. The reaction mixture consisting of 3 mL of the ABTS.+ working solution and 20 μL sample was placed in a cuvette and incubated for 5 min at 30 °C. The mixture was centrifuged (Continent 512 R, Hanil) at 2,268 × g for 5 min. The absorbance of the supernatant was measured at 734 nm using a spectrophotometer (X-ma 3100; Human Corp.). The percentage ABTS.+ inhibition was calculated as:

$${\rm{ABTSradical}}\,{\rm{cation}}\,{\rm{scavenging}}\,{\rm{activity}}\,( \% )=[({\rm{AB}}-{\rm{AS}})/{\rm{AB}}]\times 100$$

where AB is the absorbance of the blank and AS is the absorbance of the muscle extract. Trolox was used as the antioxidant standard as described in DPPH assay.

### Ferric reducing antioxidant power (FRAP) assay

As a non-radical redox potential-based method, the reducing power of each muscle extract was determined^15^. Acetate buffer (300 mM, pH 3.6), 10 mM 2,4,6-tris-2,4,6-tripyridyl-2-triazine in 40 mM HCl, and 20 mM ferric chloride solution were prepared in 10:1:1 (v/v/v) portions. The mixture was incubated in a water bath maintained at 37 °C for 30 min. FRAP (3 ml) was added to 100 μl of each sample diluted 4-fold in DDW and incubated for 5 min. The mixture was centrifuged (Continent 512 R, Hanil) at 2,268 ×*g* for 5 min. The absorbance of the supernatant was measured at 593 nm using a spectrophotometer (X-ma 3100, Human Corp.). Increased absorbance indicated greater reducing power.

### Ferrous ion chelating activity assay

The chelating activity of each muscle extract was determined by measuring the ferrous ion-ferrozine complex at 562 nm^16^. Briefly, 0.5 mL of muscle extract sample was added to 50 μl of FeCl_2_ (2 mM). The reaction was initiated by the addition of 5 mM ferrozine (0.1 ml) and 3.2 ml of ethanol. The mixture was vortexed vigorously and left in dark at room temperature for 10 min and was then centrifuged (Continent 512R, Hanil) at 2,268 *g* for 5 min. The absorbance of the supernatant was measured at 562 nm using a spectrophotometer (X-ma 3100, Human Corp.). The inhibition of ferrous ion-ferozzine complex formation was calculated as:

$${\rm{Chelating}}\,{\rm{activity}}( \% )=[1-({{\rm{A}}}_{{\rm{B}}{\prime} }-{{\rm{A}}}_{{\rm{S}}{\prime} })/{{\rm{A}}}_{{\rm{B}}{\prime} }]\times 100$$

where A_B_′ is the absorbance of the blank and A_S_′ is the absorbance of the muscle extract.

### Measurement of ACE inhibitory activity

ACE inhibitory activity of each sample was determined^17^. Ten microliters of each muscle extract was incubated with 30 μl hippuryl-L-histidyl-L-leucine (HHL, 12.5 mM in 0.1 M sodium borate buffer) at 37 °C for 10 min. Distilled water instead of sample was used as a blank and control. After incubation, 10 μl of ACE (peptidyldipeptide hydrolase from rabbit lung acetone extract) was added and the mixture incubated at 37 °C for 30 min. The enzymatic reaction was stopped by adding 50 μl of 1 N HCl. The hippuric acid generated by the action of ACE on HHL was extracted from the acidified solution into 300 μl ethyl acetate by vortex mixing for 15 s. The extract was centrifuged at 1,000 ×*g* for 5 min at 4 °C. Aliquots of 250 μL of each ethyl acetate layer were transferred to clean tubes and evaporated by heating at 70 °C for 1 h in a water bath. The hippuric acid was redissolved in 300 μl of DDW and the amount formed was determined by the absorbance at 228 nm. ACE inhibitory activity (%) was calculated as:

$${\rm{ACE}}\,{\rm{inhibitory}}\,{\rm{activity}}\,( \% )=[1-({{\rm{A}}}_{{\rm{s}}}-{{\rm{A}}}_{{\rm{SB}}})/({{\rm{A}}}_{{\rm{C}}}-{{\rm{A}}}_{{\rm{CB}}})]\times 100$$

where A_S_ is the absorbance of the sample, A_SB_ is the absorbance of the sample blank, A_C_ is the absorbance of the control, and A_CB_ is the absorbance of the control blank. Enalapril maleate salt (0.3 mg/ml) was the control.

### Protein profile using Sodium dodecyl sulfate-polyacrylamide gel electrophoresis (SDS-PAGE)

Myofibrillar proteins for SDS-PAGE analysis were prepared from unaged and crust sample using 0.03 M phosphate buffer (pH 7.4) and 0.1 M phosphate buffer potassium containing 0.7 M potassium iodide and 0.02% sodium azide as the isolating medium^18^. The protein concentration of isolated beef samples was determined and analyzed the isolated myofibrillar protein^19,20^. The stacking gel and separating gel contained 4.5% and 12.5% polyacrylamide, respectively, and 20 μl of meat extract was loaded onto the gel. Protein standards (Precision Plus Protein^TM^ Unstained Standards, Bio-Rad, Hercules, CA, USA) were included in each electrophoretic run to determine molecular size. Electrophoresis was performed using the AE-6531 Mini-Slab Size Electrophoresis System (Atto Corporation, Tokyo, Japan) at 20 mA for 70 min. Gels were stained for 30 min in 0.1% Coomassie Brilliant Blue R-250 solution containing 30% methanol and 10% acetic acid, then were destained for 90 min in 30% methanol and 10% acetic acid.

### Identification of peptides using quadrupole time-of-flight (Q-TOF) liquid chromatography tandem mass spectrometry (LC/MS/MS)

The samples (1 g) were extracted from the unaged and crust samples with 9 ml of 30% acetonitrile in deionized distilled water. The extracts were centrifuged (Combi 514R, Hanil) in Amicon^®^ ultra centrifugal filters (Ultracel^®^ - 3 K, Merck Millipore Ltd., Burlington, MA, USA) to obtain small peptides (<3 kDa). Then, the supernatants were injected to a Triple TOF 5600^®^ Q-TOF LC/MS/MS (AB Sciex, Foster City, CA, USA) using a Ultimate 3000 RSLC HPLC (Thermo Fisher Scientific Inc., Waltham, MA, USA). Mobile phase consisted of solvent A (0.1% formic acid in double-deionized water) and solvent B (0.1% formic acid in 95% acetonitrile) and the gradient programming was as follows: Solvent A and B were set at 95 and 5% from 0 to 1 min, respectively. Solvent A was decreased at 0% from 16 to 18 min. Solvent B was increased at 40% at 16 min and kept increasing at 100% from 18 to 20 min. Solvent A was increased at 95% from 20 to 26 min. Solvent B was decreased at 5% from 21 and 26 min. The flow rate was 0.3 ml/min and the injection volume was 15 μl. The auto-sampler was set at 4 °C. The electrospray ionization (ESI) mass spectra data were acquired under positive ion mode with a source temperature of 500 °C and voltage of 5500 V. The curtain gas, ion source gas 1, and ion source gas 2 were 50, 50, and 25 psi, respectively. Two full-scan mass spectra were acquired over an m/z range of 350–1800 on the MS mode. The data were collected using Analyst TF 1.7 software and analyzed using PeakView 2.2 and ProteinPilot® 4.5 (AB Sciex).

### Statistical analysis

The experimental design was completely randomized. Data were from two observations with three replications per observation. The error bars in the figures represent the mean ± standard error values (SE). Statistical analysis was performed using the SAS statistical package (version 9.3, SAS Institute Inc., Cary, NC, USA). Significant differences among the treatments were determined using the Student–Newman–Keul’s multiple comparison test at a level of *P* < *0.05*.

## Results and discussion

### Antioxidant activity

#### Radical scavenging activity

**Figure 1:**
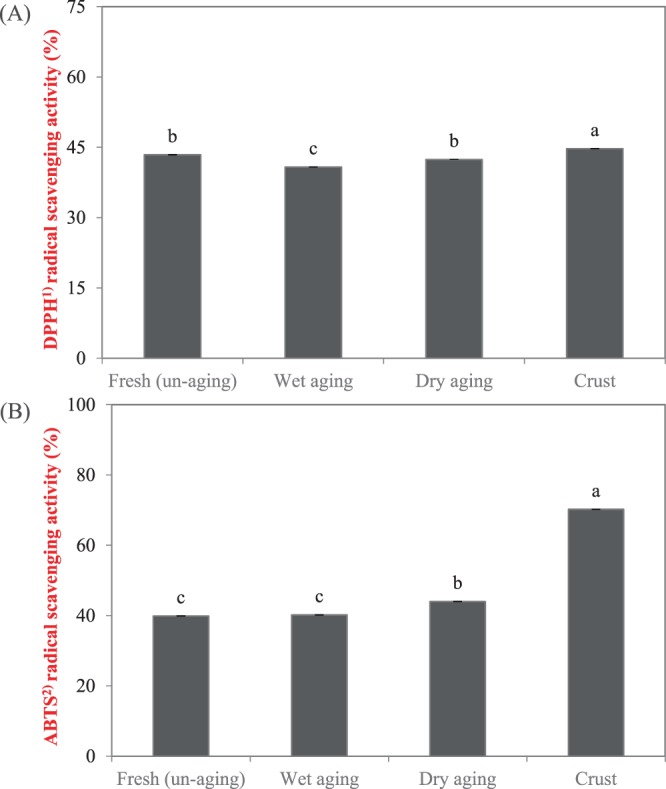
Antioxidant activity determined by radical scavenging activity of unaged, wet-aged, edible portion and crust of dry-aged beef. Error bars indicate the standard errors of means. ^a–c^Means with different letters on the bar are significantly different (*P* < *0.05*). ^(1)^DPPH (2,2-diphenyl-1-picrylhydrazyl ^(2)^ABTS [2,^2′-^azino-di-(3-ethylbenzene-thiazoline sulfonate)] radical scavenging activities.

DPPH radical scavenging activity was significantly affected (*P* < *0.05*) by the aging methods and beef portion (surface or inner) (Fig. 1(A)). Crust protein extract exhibited the greatest values (44.7% and 0.48 TEAC/ml protein extract; *P* < *0.05*) and wet-aged beef extract showed the lowest values (40.8% and 0.43 TEAC/mL extract; *P* < *0.05*) in DPPH radical scavenging activity, respectively. In this study, protein extract from dry-aged beef (flesh) also showed significantly higher value than wet-aged beef and a lower DPPH radical scavenging activity compared to that from crust (*P* < *0.05*). This result could be assumed that the presence of microorganisms^21,22^ or moisture evaporation^23,24^ produced or concentrated free amino acids during dry aging. As many studies agreed, amino acids or peptides produced by proteolytic enzymes of microorganisms present on the surface (crust) during dry aging since dry-aged beef is prone to microbial contamination and mold/yeast growth often occurs on the crust^21,22^. It is expected that both endogenous enzymes and exogenous proteolytic enzymes produced from microorganism act together, resulting in enhanced degradation of meat protein and production of different peptides in fresh, wet-aged, and dry-aged beef. It was described that naturally generated peptides by microorganisms can induce biological activity by producing peptides of different sizes and various free amino acids^25^. In addition, microbial fermentation can generate bioactive peptides having antioxidant activity from muscle-based foods^26^. Different molds and yeasts were found and reported from the crust of dry-aged beef^3,27^. In addition, during dry aging, moisture evaporation could lead to concentration of free amino acids showing antioxidant activity. Several studies reported that antioxidant activity of protein hydrolysates related to α-amino group content and properties of free amino acids or small peptides such as size, composition, etc.^28,29^. In addition, previous study reported more abundant aromatic amino acids, including phenylalanine, tyrosine, and tryptophan were present in dry-aged beef compared to wet-aged beef^23,24^. Aromatic amino acids is known as strong radical scavengers since they donate a proton to electron-deficient radicals and maintain their stability with resonance structure^30^.

The ABTS radical cation scavenging activity of four protein extracts were similar to the DPPH radical scavenging results (Fig. 1(B)). The greatest ABTS radical cation scavenging activity (69.7% and 43% higher than the lowest value) was observed in crust protein extract, followed by dry-aged beef. This also could be due to microbial growth on the crust previously described. The protein extracts from wet-aged and unaged beef displayed significantly lower ABTS radical cation scavenging activity, with no significant difference between them.

#### Ferric ion reducing power

**Figure 2:**
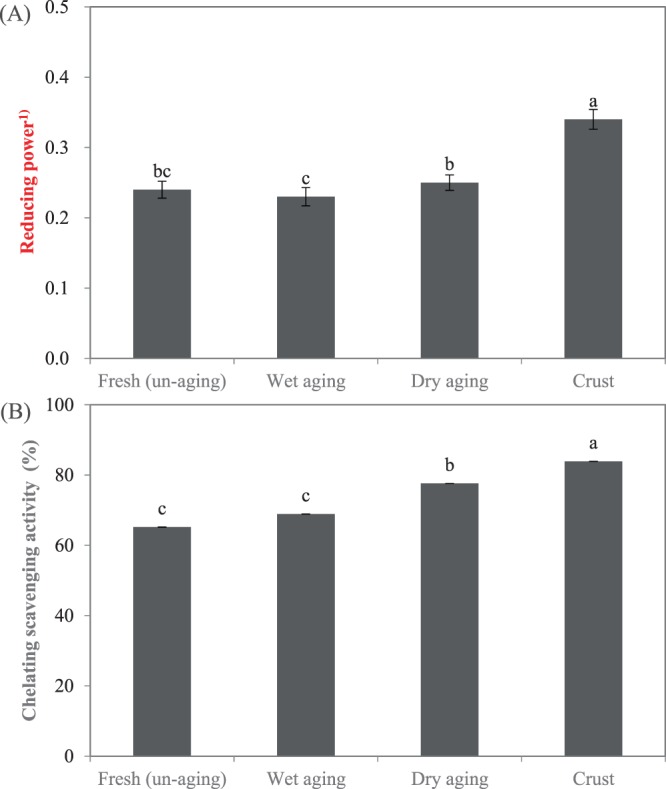
Antioxidant activity determined by (**A**) ferric ion reducing power and (**B**) metal chelating activity of unaged, wet-aged, edible portion and crust of dry-aged beef. Error bars indicate the standard errors of means. ^a–c^Means with different letters on the bar are significantly different (*P* < *0.05*). ^(1)^Reducing power measured by absorbance at 593 nm.

Reducing agents act as electron donors to the reduced species and a higher value in absorbance indicates greater reducing capacity. The highest absorbance at 593 nm (0.34; *P* < *0.05*) was observed in crust protein extract (Fig. 2(A)). Previous study presented that protein extracted from fermented sausages exhibited 2-3-time higher antioxidant activity at the end of ripening than at the initial processing stage^31^. Hence, in this study, we postulated that the direct action of microorganisms on the surface of the dry-aged meat (crust) could contribute to the generation of the reducing power capacity. The difference in absorbance among protein extracts from unaged, wet-aged, and dry-aged beef (ranging from 0.23 to 0.25) seems not to be meaningful, despite the significant difference between wet- and dry-aged beef.

#### Metal chelating activity

A clear difference in metal chelating activity was evident between the samples (Fig. 2(B)). Chelating activity was greatest for crust protein extract (83.9%; *P* < *0.05*), followed by dry-aged beef (77.6%). Chelating activities were significantly lower for protein extracts from unaged and wet-aged beef (65.2% and 68.9%, respectively). According to previous studies, dry-aged beef exhibited a higher amount of the sulfur-containing amino acid methionine^23,24^, which perhaps contributed to the increased metal chelating activity in comparison with wet-aged beef. Consequently, the type of amino acid or peptide produced by proteolysis might depend on the endogenous enzymes and those from microorganisms present on the surface of the beef during aging period.

#### ACE inhibitory activity

**Figure 3:**
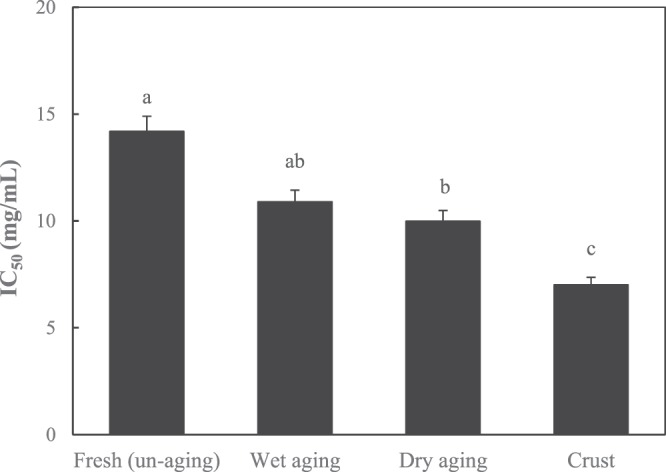
Angiotensin I-converting enzyme (ACE) inhibitory activity (IC_50_) of unaged, wet-aged, edible portion and crust of dry-aged beef. Error bars indicate the standard errors of means (1.03). ^a–c^Means with different letters on the bar are significantly different (*P* < *0.05*).

ACE inhibitory activity of protein extract was affected by the aging method (wet or dry aging) and the section of dry-aged meat [internal or external (crust) sections] (Fig. 3). Significantly high and low ACE inhibitory activities were observed for protein extract from crust and unaged beef, respectively. Similarly, it was reported that peptides derived from dry-cured ham by the action of microbial endo- and exogenous enzymes presented ACE inhibitory activity^32^. The protein extract from wet-aged beef showed similar (*P* > *0.05*) ACE inhibitory activity to unaged beef. It was described that the limited generation and/or identification of peptides with ACE inhibitory activity by endogenous proteases due to the lack of control and irregularity of endogenous proteases^33^. A recent report demonstrated that injection of thermolysin in beef loin during the marination process resulted in approximately 500-times higher ACE inhibitory activity than control (IC_50_ 1,206.0 vs 2.3 μg/mL) with an inhibitory effect on cancer cell viability after 3 days of storage^8^. Generation of small peptides through proteolysis by various enzymes (endo- and/or exogenous enzymes) increases the potent ACE inhibitory activity due to production of peptides with unspecified cleavage sites^34–36^. In addition, multi-enzymes hydrolysates derived from protein based ingredients exhibited higher ACE inhibitory activity compared to single-enzyme counterpart^37^. Generally, crust is more prone to protein decomposition by the aforementioned enzymes because the meat surface layer can be directly affected by microorganisms during dry aging. This assumption should be confirmed in a further study by investigating the effect of microorganisms (microbial enzymes) growing on the surface of beef on the production of ACE inhibitory peptides during dry aging. In addition, relationship between ACE inhibitory activity and the production of small peptide would be more discussed in Section of identification of peptides.

#### Protein profile by SDS-PAGE

**Figure 4:**
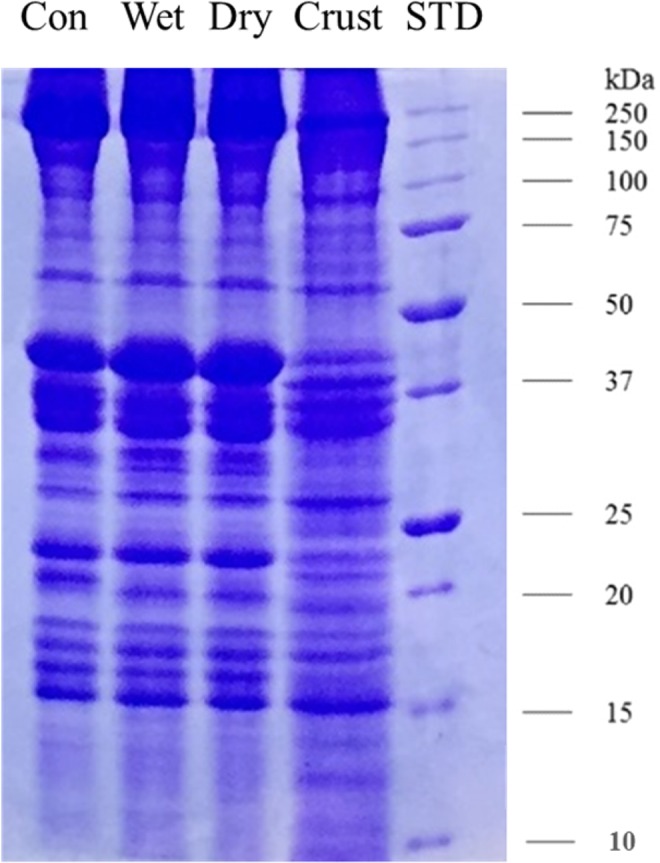
Myofibrillar protein pattern of unaged (Con), wet-aged, edible portion and crust of dry-aged beef. STD, standard molecular bands.

Protein bands with low molecular weight (<15 kDa) were more prominently observed in aged samples compared to unaged one (Fig. 4). Especially, the crust sample had higher intensity in bands with low molecular weight among the treatments. About 5–10 kDa peptides in dry sausages exhibited up to 86% of the DPPH radical scavenging activity^38^. In addition, low-molecular-weight peptides generated from protein by protease treatment exhibited higher ACE inhibitory activity compared with high-molecular-weight peptides^39^. Taken together, in this study, the generation of low molecular weight proteins may induce greater antioxidant and/or ACE inhibitory activity of crust and dry aged beef due to the proteolysis that occurs during dry aging. However, in this study, difference in appearance of protein band with <15 kDa in dry- and wet-aged samples was ambiguous. this result would be elucidated plainly based on identification of peptide and discussed in followed section.

#### Identification of peptides

Similar to the result from SDS-PAGE, much more number of small peptides (<3 kDa; 143 different types of peptides) from the aged and crust samples than those of unaged beef were detected, regardless of aging method (See Supplementary Table S1). On the other hand, types of peptides depended on aging method and location of dry-aged beef. Before aging, there were only 6 small peptides identified from the samples, while 52 and 64 different peptides from 19 and 25 different protein sources were found on the wet- and dry-aged samples, respectively. Interestingly, peptide containing same amino acid sequence was not generated between unaged and crust samples. This phenomenon was possibly due to presence of aforementioned microbial enzymes in crust which can more affected by microorganisms compared to edible portion. In our previous study, we found different changes in the metabolites of dry-aged beef by different microbial compositions^22^. Small peptides were more generated in dry-cured ham at final processing time causing by action of endopeptidase and exopeptidase by microorganisms during processing, comparing to those at initial time^40^. Many studies found that small peptides by enzymatic hydrolysis released and then it can increase functional property including antioxidant and ACE inhibitory activities in food^41^. In the present study, smaller peptides (<1 kDa) were observed in crust samples among the treatments, which was corresponded with specificity (lysyl-lysine residue; peptide No. 32) of previously reported peptide antioxidants^42^. According to previous study, peptides composing histidine at the C-terminus exhibited effective free radical scavenging activity^43^. Total 15 peptides (peptide 22, 27, 43, 51, 53, 61, 120, 137–139, 143–146, and 149) matched to aforementioned specificity were observed in all samples, except for unaged samples. In addition, hydrophobic amino acids [alanine (A), valine (V), leucine (L), and isoleucine (I)] which can effectively act to free radicals from the lipid phase as scavenger were mostly presented in meat samples of this study.

Previous studies found that substrates having N-terminal branched-chain amino acid and C-terminal hydrophobic amino acids can bind to active site of ACE as exhibiting strong affinity^44^. In this study, total 17 types of potential inhibitory peptides were observed as indicated with asterisk in Table S1. Among the 17 types of peptides, 1 (No. 125), 6 (No. 39, 40, 45, 76, 128, and 141), 7 (No. 12, 21, 38, 39, 42, 45, and 112), and 7 (No. 33, 39, 76, 95, 108, 133, and 135) types of the ACE inhibitory peptides were observed in unaged, wet- and dry-aged, and crust samples with partial overlap, respectively. ACE inhibitor possesses hydrophobic substituents at three C-terminal amino acids which can bind to active site of ACE^45^. Especially, presence of arginine (R), lysine (K) or proline (P) led to strong ACE inhibition activity in food protein-derived peptides at each of the three C-terminal position^46,47^. In this study, peptides (peptide 90 and 133) with aforementioned characteristic were observed in crust samples. Hence, the greatest (*P* < *0.05*) ACE inhibition activity in crust samples was exhibited probably due to the presence of lysine and/or proline at the three C-terminal position.

## Conclusion

Based on this study, the excellent activities of antioxidant and ACE inhibitory activities from the crust may be due to the increases in these small peptides and sequence position of functional amino acids which were generated endo- and exopeptidases. The effect of microorganisms on proteolysis and lipolysis in the crust and edible portion of meat, and the relationship with the generation of bioactive peptides and characteristic flavors are under investigation in our laboratory. Consequently, the crust could be utilized as a functional ingredient possessing antioxidant and ACE inhibitory activity instead of disuse. In other words, dry aging can be used for generation of bioactive peptides from beef loin.

## Supplementary information

Table S1.
