# Supplementary material for: Potential Antioxidant and Angiotensin I-converting Enzyme Inhibitory Activity in Crust of Dry-aged Beef
Source: Sci Rep. 2020 May 12;10:7883. doi: 10.1038/s41598-020-64861-0 (PMC7217845; doi:10.1038/s41598-020-64861-0)
Supplement: Supplementary file 1 — Table S1. [file 41598_2020_64861_MOESM1_ESM.pdf]

Potential Antioxidant and Angiotensin I-converting Enzyme Inhibitory Activity in Crust of Dry-aged Beef

Juhui Choe[1], Bumjin Park[1], Hyun Jung Lee[1], Cheorun Jo [1,2,*]

[1] Department of Agricultural Biotechnology, Center for Food and Bioconvergence, and Research Institute of Agriculture and Life Science, Seoul National University, Seoul 08826, Korea

[2]Institute of Green Bio Science and Technology, Seoul National University, Pyeongchang 25354, Korea

***Correspondence:** Cheorun Jo, Department of Agricultural Biotechnology, Seoul National University, Seoul 08826, Republic of Korea.

Tel.: +82-2-880-4804; Fax: +82-2-873-2271; E-mail: cheorun@snu.ac.kr

Table S1. Small peptides (3 kDa) identified by LC/MS/MS from un-aged, wet-aged, dry-aged, and the crust of beef sirloins

**Protein name**	**No.**	**Peptide sequence**	**Observed MW (Da)**	**Aging method/portion**			
				**Non-aged (Fresh)**	**Wet aging**	**Dry aging**	**Crust**
Actin, alpha cardiac muscle 1	1	AGFAGDDAPR	975.44				+
	2	AVFPSIVGR	944.55				+
	3	DNGSGLVKAGFAGDDAPR	1745.84				+
	4	FPSIVGR	774.44				+
Actin, alpha skeletal muscle	5	DEDETT	750.25			+	
	6	ITKQEYDEAGPSIVHRK	1970.04		+	+	
	7	TKQEYDEAGPSIVHRK	1856.94		+		
Alpha-1,4 glucan phosphorylase	8	MPAPDEKI	899.44				+
	9	SRPLTDQEKRKQISVRG	2039.13			+	
AP complex subunit beta	10	LGDLLNLDLGPPVSG	1478.88			+	
	11	TVEISLP	757.39			+	
AP-5 complex subunit beta-1	12	LNPASGASGRLLPLL*	1478.89			+	
	13	LPLAGD	584.35			+	
Arf-GAP with SH3 domain, ANK repeat and PH domain-containing protein 1	14	DPPSPLP	721.44		+		
ATF6B protein	15	LGDDPTSP	800.40		+		
Beta-catenin-like protein 1	16	SIAVVDL	715.39			+	
BTB/POZ domain-containing protein KCTD15	17	RSPVSPL	754.42			+	
Carbonic anhydrase	18	IVKASFK	791.49				+
Carbonic anhydrase 3	19	NWRPPQPIKGR	1348.74				+
Cardiac phospholamban	20	MDKVQYLTR	1194.61				+
Cbp/p300-interacting transactivator 1	21	LPELWL*	769.44			+	
Coagulation factor V	22	EVGDIMKVH	1026.57				+
Creatine kinase M-type	23	DDVIQTG	746.35			+	
	24	DEESYTVFKD	1231.53		+	+	
	25	DKPVSPL	754.42		+	+	
	26	DPIIQDR	855.45				+
	27	FKAEEEYPDLSKHNNH	1956.91				+
	28	GVDNPGHPF	938.43				+
	29	IDDHFL	758.36				+
	30	PFGNTHNK	913.45				+
	31	SIDDMIPAQK	1116.55				+

	32	TLEIYKK	893.53			+
	33	VVDGVKL*	728.44			+
CTF18, chromosome transmission fidelity factor 18 homolog	34	PTLDITPP	852.49	+		
Diacylglycerol kinase	35	IPVP	424.27	+		
	36	PAPEPAPVP	873.46	+		
DKK3 protein	37	PVCTPLPVEG	1026.56		+	
Elastin	38	AKLGAGGAGVLP*	1009.63		+	
	39	AVGIGGIP*	682.44	+	+	+
	40	VGIGGIP*	611.40	+		
	41	VGVGG	387.21			+
	42	VIGAGVP*	611.40		+	
Fructose-bisphosphate aldolase	43	ESLFISNH	946.44			+
	44	ESLFISNHAY	1179.56			+
Glyceraldehyde-3-phosphate dehydrogenase (GAPDH)	45	AVGKVIP*	682.44	+	+	
	46	AVGKVIPE	811.48			+
	47	AVGKVIPELN	1038.61			+
	48	AVGKVIPELNGK	1223.73			+
	49	AVGKVIPELNGKLT	1437.86	+	+	
	50	AVGKVIPELNGKLTG	1494.88	+		
	51	DAGAGIALNDH	1052.48	+		
	52	DAGAGIALNDHFVK	1426.73	+	+	
	53	DFNSDTH	834.31	+	+	
	54	DNEFGYSNR	1100.45	+	+	
	55	DPANIKW	842.43		+	
	56	EKPAKYDEIKKVVKQASEGPLK	2484.40	+		
	57	ELNGKLT	773.43		+	
	58	FDAGAGIALNDHFVK	1573.79	+	+	
	59	GAAKAVGKVIP	1009.63		+	
	60	GFGRIGR	761.43			+
	61	GIALNDH	738.37	+	+	
	62	GIALNDHFVK	1112.60	+	+	
	63	IALNDHFVK	1055.58	+		
	64	IIPASTGAAKAVGKVIP	1591.97		+	
	65	IPASTGAAKAVGKVIP	1478.89		+	
	66	IPASTGAAKAVGKVIPELN	1835.05	+	+	
	67	IPASTGAAKAVGKVIPELNGKLT	2234.31	+	+	
	68	IPASTGAAKAVGKVIPELNGKLTG	2291.32	+	+	

	69	IPELN	584.32	+	+	
	70	IPELNGK	769.43	+	+	
	71	IPELNGKLT	983.56		+	
	72	NDHFVK	759.36		+	
	73	NDHFVKL	872.44		+	
	74	SSTFDAGAGIALNDHFVK	1848.91	+	+	
	75	TGAAKAVGKVIP	1110.68	+	+	
	76	VGKVIP*	611.40	+		+
	77	VGKVIPELN	967.57			+
	78	VGVNGFGR	804.43			+
	79	VGVNGFGRIGR	1130.64			+
	80	VPTPNVS	712.37	+	+	
	81	VPTPNVSVVD	1025.54			+
	82	VPTPNVSVVDLT	1239.67		+	
G-protein coupled receptor family C group 6 member A	83	AASPGHIM	782.34		+	
GSTM1 protein	84	LAVWGNK	786.44			+
Histone acetyltransferase	85	PPQPQPPAPPPP	1218.65	+		
Importin subunit alpha	86	QQQVQAVIDAGLIP	1478.88		+	
Matrix metallopeptidase 19	87	LPTIPLVP	848.54	+	+	
Meiosis arrest female protein 1	88	KDVPSPL	754.42	+		
	89	LPLK	469.32	+		
MYBPC1 protein	90	FKRSGEGQDDAGELDFSG LLKRR	2580.32			+
	91	PDPP	424.23			+
Myoglobin	92	DMAAQYKVLGFHG	1435.69	+		
	93	GGILK	486.32			+
	94	TALGGIL	643.39			+
	95	VAGHGQEVL*	908.47			+
	96	VLGFHG	628.34			+
Myosin light chain 1/3	97	AAPAPAPAPAPAPAPP KEEK	2045.10			+
Myosin, light chain 6B	98	ELPSL	557.30			+
	99	IPVILEKPAK	1106.71			+
	100	PAVGPPPSR	876.48			+
	101	VDAEM	605.23			+
	102	VGPPPSR	708.39			+
Myozenin-1	103	NRTPIP	697.38		+	
	104	NVDISIP	757.39		+	
	105	YNVDISIPLD	1147.58		+	

Nuclear receptor binding factor 2	106	DIPIPNLPP	974.48			+	
O(6)-methylguanine-induced apoptosis 2	107	PAANAYT	706.46				+
Olfactory receptor	108	LGNLGLIL[*]	811.48				+
	109	LILLIWVD	983.56		+		
Paternally-expressed gene 3 protein	110	KAAGASSLSAPPAA	1197.71		+		
PDZ and LIM domain 5	111	DWHHEVS	908.38				+
Phosphodiesterase	112	LLADPSLP[*]	824.46			+	
Phosphoglycerate kinase 1	113	VGVN	387.21				+
	114	AAVPSIK	684.42				+
	115	ALESPER	800.41				+
	116	SLSNKLTLDKLDVKGK	1800.04				+
PIH1 domain-containing protein 1	117	VGENR	573.30				+
PIM2 protein (Fragment)	118	PMTPTPLP	852.49		+	+	
Plastin-3 OS=Bos taurus GN=PLS3 PE=1 SV=1; Plastin-3	119	SLAVVDL	715.39		+		
Probable cystatin-15	120	PLLLGLLALGPH	1212.74			+	
Programmed cell death protein 2	121	PSEDPPSE	856.39			+	
Protein YIPF6	122	EGEITIP	757.39			+	
Pyruvate kinase	123	FTNTMRVVPVP	1259.67	+	+	+	
	124	NTMRVVPVP	1011.55	+			
RBM34 protein	125	LQPVYVPVP*	1011.55	+			
Retinoic acid receptor RXR-gamma	126	GINLVAP	682.44				+
RING finger protein 10	127	ALSLSPLSR	942.53		+		
Ring finger protein 214	128	VRNGAKLSSLPQIP[*]	1478.88		+		
Sodium channel protein	129	PEVEEVPVEQ	1154.52		+		
Solute carrier family 31 (Copper transporters), member 2	130	PVSRSPL	754.42			+	
Target of rapamycin complex subunit LST8	131	IPEPEVSIT	983.57			+	
Targeting protein for Xklp2	132	SGSLVQEP	815.44			+	
TOX high mobility group box family member 4	133	ASMQLPPP[*]	855.44				+
	134	IVPP	424.23				+
	135	LVPP[*]	424.23				+
Transcription initiation factor IIB	136	PECGLVVG	772.36	+			
Troponin T fast skeletal muscle type	137	AQEEAPPPPAEVPEVHEEVH	2190.03			+	
	138	EAPPPPAEVPEVHEEVH	1861.89		+	+	
	139	EEAPPPPAEVPEVHEEVH	1990.94		+	+	

	140	SDEEVEHVE	1113.45			+	
	141	APPPPAEVP*	873.46		+		
	142	APPPPAEVPEVHEE	1496.71		+		
	143	APPPPAEVPEVHEEVH	1732.84		+	+	+
	144	APPPPAEVPEVHEEVHEVH	2098.01		+		
	145	EVPEVHEEVH	1202.56		+	+	
	146	VPEVHEEVH	1073.52		+	+	
UBA domain containing 1	147	PTIDTPLP	852.49	+	+	+	
UPF0609 protein C4orf27 homolog	148	GAGLVVP	611.40		+		
Vasopressin V1b receptor	149	MQVFVLH	872.44			+	

+ indicates the presence of identified peptides from each treatment sample.

Small peptides which consists of an branched-chain amino acids at the N-terminus and hydrophobic amino acids at the C-terminus are indicated with an asterisk (*).
